# Effects of (Oxy-)Fluorination on Various High-Performance Yarns

**DOI:** 10.3390/molecules21091127

**Published:** 2016-08-26

**Authors:** Iris Kruppke, Matthias Bartusch, Rico Hickmann, Rolf-Dieter Hund, Chokri Cherif

**Affiliations:** Institute of Textile Machinery and High Performance Material Technology, Technische Universität Dresden, Hohe Straße 6, 01062 Dresden, Germany; matthias.bartusch1@tu-dresden.de (M.B.); rico.hickmann@tu-dresden.de (R.H.); rolf-dieter.hund@tu-dresden.de (R.-D.H.); chokri.cherif@tu-dresden.de (C.C.)

**Keywords:** (oxy-)fluorination, carbon fiber, ultra-high-molecular-weight polyethylene, polyphenylene sulfide, polyphenylene terephthalamide

## Abstract

In this work, typical high-performance yarns are oxy-fluorinated, such as carbon fibers, ultra-high-molecular-weight polyethylene, poly(*p*-phenylene sulfide) and poly(*p*-phenylene terephthalamide). The focus is on the property changes of the fiber surface, especially the wetting behavior, structure and chemical composition. Therefore, contact angle, XPS and tensile strength measurements are performed on treated and untreated fibers, while SEM is utilized to evaluate the surface structure. Different results for the fiber materials are observed. While polyethylene exhibits a relevant impact on both surface and bulk properties, polyphenylene terephthalamide and polyphenylene sulfide are only affected slightly by (oxy-)fluorination. The wetting of carbon fiber needs higher treatment intensities, but in contrast to the organic fibers, even its textile-physical properties are enhanced by the treatment. Based on these findings, the capability of (oxy-)fluorination to improve the adhesion of textiles in fiber-reinforced composite materials can be derived.

## 1. Introduction

In the field of lightweight construction, fiber-reinforced composite materials are widely used. Due to their low specific weight, their excellent chemical and physical resistance, high-performance textiles are more and more established instead of classic, i.e., metal constructions. Additionally, they can be used to produce structures with different and customized geometries [1].

By selecting a specific fiber type with its unique chemical and structural composition, a great variety of properties can be achieved: e.g., carbon, glass and ceramic fibers exhibit the highest temperature resistance along with a high stiffness. Furthermore, poly(*p*-phenylene sulfide) (PPS), poly(*p*-phenylene terephthalamide) (PPTA) and polyether ether ketone (PEEK) have high operating temperature and are more flexible due to their polymeric chain structure. Additionally, organic fibers have lower densities than fibers based on inorganic materials. Ultra-high-molecular-weight polyethylene (UHMW PE), on the other hand, has low melting temperatures, but possesses one of the highest specific strengths among all technical textiles and, like most of the man-made fibers, exhibits high chemical inertness and also low to almost nil water adsorption [2].

One of the essential challenges for the use of high-performance fiber is the sufficient adhesion to typical matrix materials. The interfacial interactions between the reinforcing fibers and the matrix are decisive and have to be improved without altering the bulk properties of the textile. The classical fibers, such as UHMW PE, PPTA, PPS or PEEK, as well as inorganic fibers based on glass, basalt or carbon, exhibit only a few opportunities for chemical interactions due to their chemical structure. During manufacturing, fibers are additionally coated with special sizings to provide good textile handling properties and reduced damages to fiber. Unfortunately, these lead to a reduced adhesion to polymeric matrix systems.

To enhance surface interactions, customized sizings are applied to almost all commercial fibers used in the field of textile-reinforced plastics. Such sizings can be used to provide a better adhesion to the polymer matrix by introducing functional groups that are not available on the fiber material themselves. This is the case for carbon and glass fibers. However, since sizings are only held by adhesive forces, they are prone to degradation and removal by mechanical stress or chemical impact. Consequently, further surface treatments are necessary to improve the surface properties of, e.g., carbon fibers (CF).

Established processes for the surface activation of polyolefins are various kinds of plasma treatments under reduced or atmospheric pressure, with and without additional grafting of functional polymers [3,4,5,6]. Most of these principles are also suitable for PPTA [7,8], PPS [9,10] and CF [11,12]. The most significant disadvantage here is the limited stability of plasma treatment over time. The penetration depth of the surface alterations ranges from 10 up to 100 nm [13]. Therefore, the functional groups tend to diffuse into the substrate by polymer chain movement to minimize the surface energy [14]. Wet-chemical treatments are of less interest, since they are energy-intensive and often not environmentally friendly, due to the chemicals used and the water wastage. Nevertheless, it is still common to pre-treat carbon fibers prior to the application of sizing by wet-chemical treatments, such as using nitric acid [15,16,17], sulfuric acid [18], potassium nitrate [19,20] and methanesulfonic acid [17]. In addition, electro-chemical treatments are also known, e.g., with nitric acid, potassium nitrate [20,21] and phosphoric acid [22] or trifluoroacetic anhydride vapor [23].

Other methods also under investigation include radiation starting from visible and UV light up to alpha, gamma and electron radiation [24]. Through high-energy radiation, radicals are created, which undergo various reactions depending on the process conditions [25,26]. Electron beams can be used to selectively cross-link polymer chains, while laser light is used to roughen the surface by ablation of material in a micrometer scale, which leads to an increase of the total surface area and of the mechanical interactions.

Another method of interest for surface activation is the (oxy-)fluorination. Basically arising from polyolefin treatment [27], fluorination of polymers has been long known to be very effective for barrier properties. The (oxy-)fluorination, i.e., the addition of oxygen sources (moisture, air, oxygen, etc.), is known to enhance the surface functionality by an increase of the polarity of the substrate [28,29,30]. The use of (oxy-)fluorination in the field of technical textiles has been studied at this given time. For carbon fibers, research has been done by Park et al. [31,32,33,34]. Furthermore, various technical textile polymers were oxy-fluorinated to enhance their surface properties [35,36,37].

In principle, elemental fluorine is easily dissociated under ambient conditions, forming highly reactive fluorine radicals. In contrast to organic compounds, a hydrogen-abstraction is taking place under the formation of gaseous hydrogen fluoride, and free electrons are transferred to the substrates’ surface. From this point, available fluorine radicals and reactive agents can be bound to the surface. In the case of a gaseous reaction mixture with air, oxygen-rich functional groups can be introduced.

The (oxy-)fluorination process possesses various benefits. Firstly, the energy consumption is low, since the reaction occurs at room temperature. Secondly, the reaction takes place in gaseous form, which on the one hand, allows the fluorine to penetrate into almost any open geometry without requiring special processing steps. On the other hand, no further washing, drying or other steps are necessary subsequent to the (oxy-)fluorination due to the complete absence of water or other solvents. Since radical reactions occur during (oxy-)fluorination, chemical and physical changes of the treated fiber surfaces are to be expected. The determination of the wetting behavior by means of contact angle measurement and XPS analysis reveals differences in the outmost layer of the substrate, while SEM imaging gives information about the physical structure of the surface. Tensile strength tests are used to ensure the integrity of the bulk material and to evaluate the textile-mechanical properties, which are most relevant for the utilization as reinforcing fibers.

## 2. Results and Discussion

### 2.1. Contact Angles and Surface Energies

**Figure 1:**
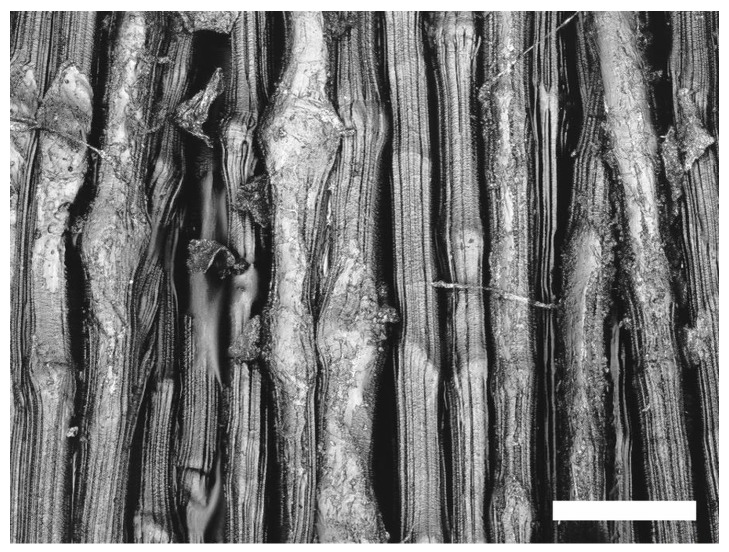
Images of treated UHMW PE fiber; bar = 50 μm.

The (oxy-)fluorination shows different efficiencies depending on the chemical structure of the substrate. The results are presented in Table 1 UHMW PE exhibits both increased polar and disperse surface energies, resulting in an increased wetting with both hydrophilic and hydrophobic liquids. A similar behavior can be observed for the carbon fiber, albeit in a less distinct manner by a factor of 2.3 for the polar part, although significantly higher treatment intensities are necessary here. In this case, the total surface energy of the PE fiber increases by 48% and the carbon fiber by 19%. Likewise, intensities applied onto PE led to strong macroscopic destruction of the bulk material due to cross-linking and degradation reactions as shown in Figure 1.

Treated PPS exhibits increased polar surface energies up to 12.0 mN/m, indicating better wetting by hydrophilic liquids, but looking at the contact angles against water, it is obvious that only a slight decrease of the contact angle can be achieved here. Thus, the main impact arises from the strongly increased contact angles against diiodomethane. This leads to the conclusion that the total surface interactions are reduced, in numbers by 23% (total surface energy), probably due to partial fluorination of the surface, although the fiber in total exhibits a slightly more hydrophilic behavior.

**Figure 2:**
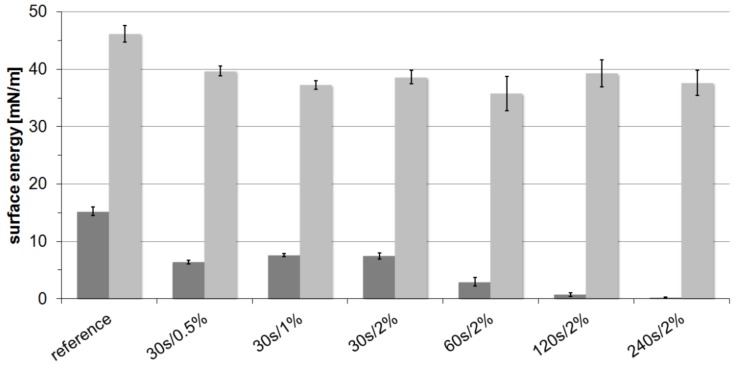
Surface energies of PPTA yarn samples oxy-fluorinated with different treatment times and fluorine content; dark grey, polar surface energy; bright grey, dispersed surface energy.

In contrast, PPTA shows increased contact angles against both testing liquids. Further experiments (Figure 2) also revealed that increased treatment times lead to a continuous increase of contact angles against water and therefore reduced polar surface energies. However, the variation of fluorine content shows only a negligible impact on the hydrophilicity, and the dispersed part always exhibits values of around 38 mN/m independent of the treatment conditions. Consequently, the total surface energy for PPTA is reduced by 35%.

In the literature, it can be found that C-F groups are introduced by gas-phase fluorination of aramid fibers [38], while only small amounts of additional oxygen groups are introduced in the presence of oxygen or water during the treatment [37]. It is obvious that due to the chemical structure of both PPTA and PPS, mostly aromatic C-H structures are attacked by fluorine radicals. It can be assumed that the further reaction with oxygen is quite slow in comparison to the fluorination reaction, as well as in comparison to the reaction at aliphatic C-H structures, at which the radicals are not stabilized by *π*-electron systems.

### 2.2. Scanning Electron Microscopy

**Figure 3:**
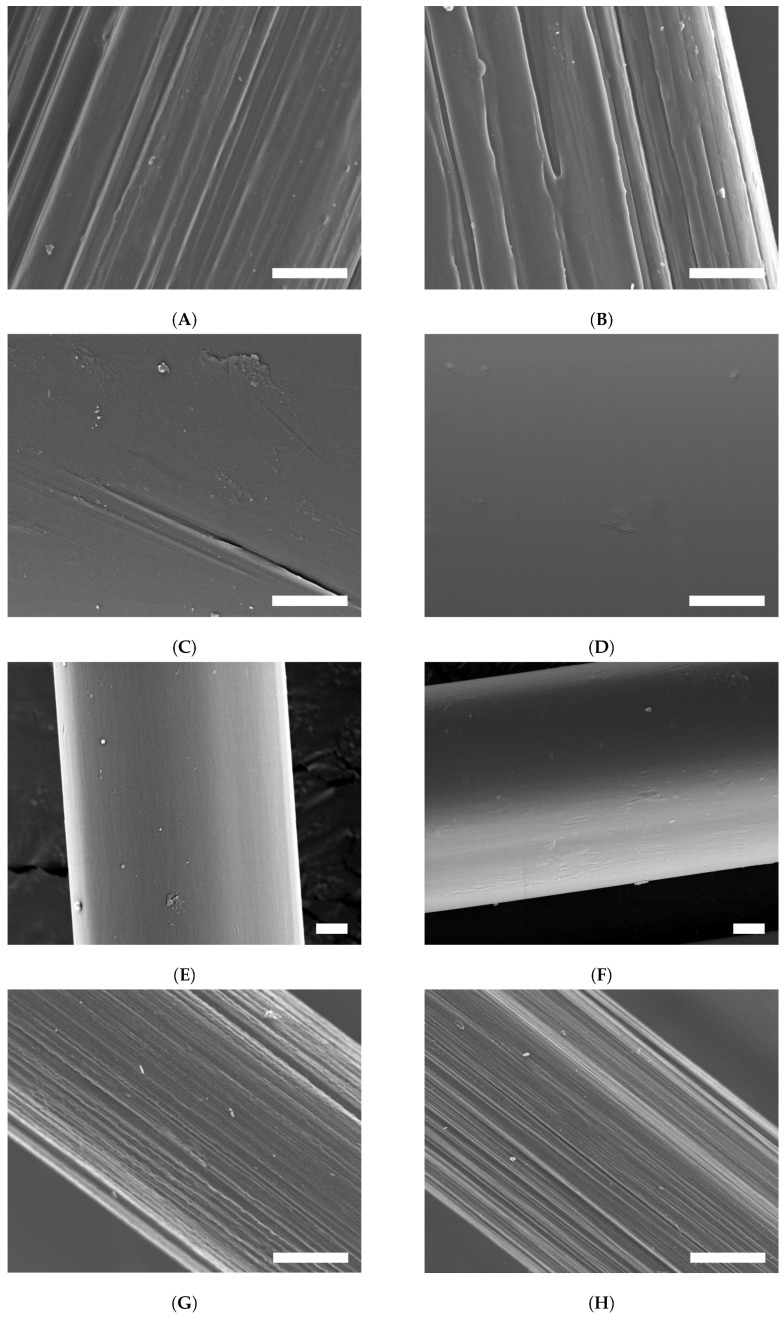
SEM images of treated and untreated fibers; bar = 2 μm. (**A**) untreated UHMW PE; (**B**) treated UHMW PE; (**C**) untreated PPS; (**D**) treated PPS; (**E**) untreated PPTA; (**F**) treated PPTA ; (**G**) untreated CF; (**H**) treated CF.

The surface structure revealed by scanning electron microscopy shows only slight visible changes at higher magnification (Figure 3). The surface of UHMW PE fiber (A) typically consists of individual fibrils, sporadically distorted by irregularities arising from the drawing process during manufacturing. After (oxy-)fluorination (B), the surface profile of the fibrils became sharpened, exhibited less fragments and appeared more closed. Additionally, this surface is roughened by ablation, which leads to grooves perpendicular to the fiber axis, similar to surface structures after atmospheric pressure plasma [3].

The untreated PPS fiber sample (C) shows a very smooth surface with nearly no remarkable structures of the fiber visible. In comparison, the surface after the fluorination (D) looks even smoother than the untreated one. The fluorination does not lead to a surface roughening or fiber defects.

SEM images of PPTA (E) reveal a smooth surface with little particles on it, which may be out of the desizing-process. After fluorination (F), the surface shows a more rough structure. This is a sign that the treatment leads to the abrasion of the PPTA fiber. A possible advantage could be a higher adhesion out of this rough surface. A loss of fiber strength due to the surface ablation could not be observed.

Carbon fibers exhibit typical striations of stretching the fibrous polyacrylonitrile precursor, as shown in Figure 3. The desized carbon fiber (G) presents a scarified structure and intense imperfections, as well as small particles. After the (oxy-)fluorination process (H), there can be seen a change in surface morphology in the micrometer range. The fibrous structure becomes more obvious by the etching and flattening of the (oxy-)fluorination process, which was also shown by Park et al. [31]. Small tears and finer structures become visible. The resulting roughness provides furthermore a better interfacial adhesion.

### 2.3. X-Ray Photoelectron Spectroscopy

As to be expected, all of the untreated fibers show no fluorine in their structure. After (oxy-)fluorination, significant amounts of fluorine ranging from 4.0% up to 18.0% can be found in all samples (Table 2). While UHMW PE and PPS fibers also have significant amounts of oxygen integrated, PPTA and CF exhibit only slight increases of oxygen concentration compared to their references.

In the wide-scan spectrum and in the C1s peak of desized UHMW PE, sp^3^ hybridized carbon is almost exclusively found, originating from the CH${}_{2}$ backbone. It accounts for 97.1% of the peak at 285.0 eV. With 2.6%, only small amounts of oxygen are detected most likely remnants from the sizing or contaminations attached to the fiber surface. The C1s peak exhibits a slight tailing originating from carboxylic groups (C-C(=O)O- at 285.9 eV and -C(=O)O- at 289.4 eV) and -C-O- structures at 286.7 eV.

After (oxy-)fluorination, the C1s peak broadens to higher electron energies up to 290 eV, showing increased amounts of carboxylic groups, as well as -C-O- structures. The total concentration of oxygen rises to 13.1% in the wide-scan spectrum, resulting in a C:O ration of 0.176. At 287.6 eV, around 8.4% of the C1s peak are contributed by -C-F, and at 290.2 eV, another 3.7% from the -C-F${}_{2}$ groups can be observed. In total, almost 12% of fluorine are integrated into the UHMW PE leading to a C:F ratio of 0.157. The drastic change in the wetting behavior is therefore explained by the high amount of newly-created functional groups at the fiber surface. Both before and after (oxy-)fluorination, no nitrogen was found in the outer polymer layer.

The XPS analysis of pristine PPS exhibits a quite high amount of oxygen (9.8%) and a low amount of sulfur (3.5%) compared to the theoretical structure. This could be explained by partial oxidation of the sulfur to sulfoxide and further to sulfone [39], although no shift of the S2p double peak at 163.8 (S2p 3/2) and 165.0 eV (S2p 1/2) was observed. Since the C1s peak at 285.0 eV also shows various oxygen containing groups at higher energies up to 289.0 eV, it can be assumed that an oxygen-rich layer exists on the fiber surface maybe due to some sizing left. The largest component of these groups arises from -C-O- at 286.6 eV with 11.6% of the C1s peak.

After the (oxy-)fluorination process, a significant amount of fluorine (13.6%) can be found in the PPS surface. Additionally, the C:O ration increases from 0.115 to 0.215, resulting in an increased concentration of the -C-O- (12.6% at 286.7 eV) and -C(=O)- groups (10.6% at 287.6 eV). Furthermore, the broadening of the C1s peak up to 290.9 eV indicates the presence of the -C-F and -CF${}_{2}$ groups. The sulfur concentration decreases slightly to 2.3% in the surface, and additionally about 0.7% of nitrogen are inserted into the fiber. Although around 50% more oxygen is found in the PPS after (oxy-)fluorination, the wetting behavior against water increases only slightly, while the adhesion of diiodomethane is clearly enhanced.

For desized PPTA fiber, the XPS spectrum exhibits an atomic concentration of carbon of 76.6%. At 284.4 eV, the largest component results from sp^2^ hybridized C atoms in the aromatic ring structures of PPTA. Furthermore, the large tailing to higher energies indicates the presence of conjugated *π*-electrons. At 285.5 eV and 287.5 eV, the two carbon atoms in the amide group (-C(=O)- and -C-NH-) can be observed. With a relation of 5:1:1, the atomic concentrations correspond to the expected ratio of the theoretical PPTA structure. Additionally, the spectra reveal other peaks at 285.0 eV, 286.2 eV and 287.4 eV, which are probably caused by functional groups of remnants after the desizing. The appropriate structures are sp^3^ hybridized carbon, ether groups (C-O-C) and carbonyl groups, respectively.

As shown, around 15.6 at% fluorine are introduced into the surface of oxy-fluorinated PPTA fiber, which results in almost the same amount as of oxygen, since its content only rises slightly from originally 15.2% to 16.3%. The conjugated *π*-electron system seems to be degraded by the radical reactions. As a result, the concentration of sp^2^ hybridized carbon atoms decreases from 49.0% to 33.0%. Furthermore, the number of sp^3^ hybridized carbons increases from 8.2% to 10.8%. As it is known, a complete fluorination of an aromatic ring, including degradation of the *π*-electron system, is possible, e.g., for poly(p-phenylene), if there is a higher reaction temperature and no other reactants [40]. In our case, the presence of oxygen, as well as a lower process temperature lead to a partial hydrogen substitution.

In comparison to the theoretical structure, the number of amide groups is reduced from 9.8% to 5.8%, which corresponds to the smaller element concentration of nitrogen measured after the (oxy-)fluorination (7.5% to 2.7%). Fluorine is incorporated in the form of various groups, e.g., -C-F and -CF${}_{2}$, resulting in additional peaks between 287.0 eV and 290.6 eV and a total amount of 30.6% of carbon atoms. In combination with the small amount of oxygen added into the PPTA structure, this explains the loss of hydrophilicity.

For the XPS investigation, an untreated and desized carbon fiber was compared with a desized and oxy-fluorinated sample. The elemental analysis exhibits a changed surface composition after the (oxy-)fluorination. The original surface composition was characterized by the carbon fiber itself, as well as the thermal desizing. Therefore, about 13.6% O1s and 3.0% of N1s were observed for the reference fiber.

After the treatment, the concentration of nitrogen decreases to 2.1%. This can be seen in the C:N ratio in Table 2 resulting in a slight decrease from 0.037 to 0.027. The oxygen concentration stays almost the same, increasing only from 13.6% to 13.9% after (oxy-)fluorination, which results in a final C:O ratio of 0.175. Furthermore, the spectrum exhibits the typical shift of an oxidized carbon peak compared to a graphite peak [41].

Before the treatment, typical -C=O bonds are observed at 288.2 eV, which is a result from the oxidation during thermal desizing. Around 22.9% and 65.9% of the C1s peak are contributed by sp^3^ and sp^2^ hybridized carbon, respectively. A small amount of 3.5% is related to conjugated *π*-electrons. After (oxy-)fluorination, the measurable fluorine content is 3.96% (F1s). Fluorine-related XPS peaks within the C1s peak are observed: at 289.9 eV, 4.0% of the total C1s peak can be related to CF${}_{x}$-groups; at 288.2 eV, both -C=O and CF-CF contribute to the increased concentration of 8.4% of the C1s peak compared to the reference. Although the (oxy-)fluorination was more intensive compared to the other fibers, the incorporated fluorine content is still low. The C:F ratio rises only to 0.050 (Table 2). Furthermore, a slight increase of oxygen is found. Eleven-point-five percent of the C1s peak result from C-O-groups at 286.5 eV in addition to the peak at 288.2 eV. The concentration of sp^3^ hybridized carbon decreases to 11.6%, as well as the conjugated *π*-electron-systems (0.8%).

**Figure 4:**
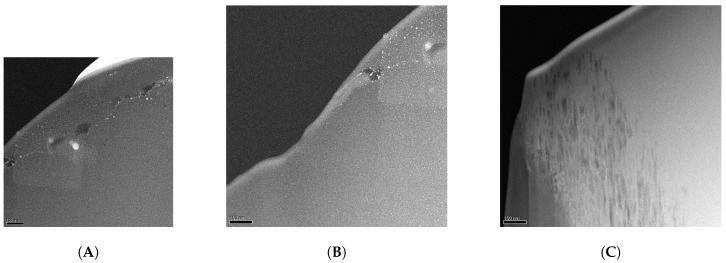
TEM dark field image (cross-section) with two areas of interest: region of interest A (ROI A) and region of interest B (ROI B) at the edge of the carbon fiber in the low-loss region of untreated carbon fiber (**A**,**B**) and the edge of treated carbon fiber (**C**). (A) Region A; (B) Region B; (C) treated CF.

**Figure 5:**
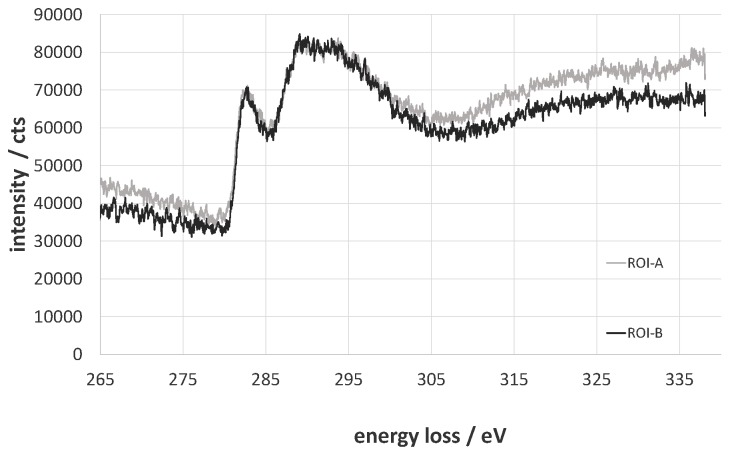
Electron energy-loss near the edge structure of the C-edge of ROI A and ROI B.

The total amount of carbon decreases from 82.1% to 79.4% for the oxy-fluorinated sample. According to this, the concentration of sp^2^ hybridized carbon changes from 65.9% to 63.7% and can be seen as retained [42]. It was shown by A. Hamwi [43] that the accretion of fluor at room temperature occurs mainly at amorphous carbon surfaces with a low number of graphitic structures and in the presence of HF or other volatile fluorine compounds. The used carbon fiber exhibits an amorphous structure at the edge region (Figure 4A,B), which could be shown via TEM/EELS by typical plasmon peaks of *π*-bindings at 283 eV and a broad σ* peak (Figure 5, ROI A and ROI B) [44,45]. Typically, two plasmon peaks were observed, and the received spectra are related to those of amorphous carbon. After (oxy-)fluorination treatment, the carbon fiber became more amorphous in the outer layer in the dimension of 500 nm, as shown in Figure 4C. It can be assumed that the change of the hybridization of carbon took place in that exposed area. This leads to the assumption of a changed bulk structure of carbon fiber by (oxy-)fluorination. In conclusion, the (oxy-)fluorination still introduces surface functional groups based on oxygen, which improve the surface functionality and the resulting adhesion behavior. Fluorine-containing groups are also established.

### 2.4. Tensile Strength Tests

For the tensile strength tests, again, different results are observed for the various fiber materials (Table 3). The UHMW PE fiber is obviously stiff damaged during the treatment by degradation reactions as already found after (oxy-)fluorination with high intensities. Therefore, breaking force and tensile stress are reduced by about 13% under the applied treatment conditions. Maximum elongation is reduced, as well, probably due to cross-linking. Considering its application as high strength reinforcing fiber, a compromise between the surface activation and loss of tensile strength should be found by decreasing the treatment intensity to ensure sufficient textile-physical properties are achieved.

The bulk structure of PPS fiber is only slightly influenced by (oxy-)fluorination. A decrease of about 2.5% in tensile strength is observed. Therefore, it can be said that this is mainly unchanged with regard to both bulk and surface mechanics.

The stress-strain behavior of PPTA fiber is not significantly altered by the (oxy-)fluorination. The maximum breaking force is almost identical before and after treatment. It can be assumed that the (oxy-)fluorination leads to no negative effects, and the excellent mechanical properties of the PPTA are retained after the treatment.

For the non-treated condition, the used CF exhibits a comparably low tensile strength of 1.3 GPa. Nevertheless and in contrast to the other investigated high-performance fibers, the oxy-fluorinated carbon fibers show an increase for breaking force and elongation of about 15.4% and 12.6%, respectively. The formation of covalent -C-F- bonds avoids the migration of fluorine into the graphite structure of carbon fibers at low temperatures [42]. The strengthening effect may result by the substitution of C-atoms by fluorine and the novel amorphous outer layer as mentioned for the XPS results, which can induce a harder area, as known for ceramics. Therefore, on the other hand, the covalent (CF)${}_{n}$ groups and the intercalated fluorine in carbon fibers have a positive influence on the respective physical and mechanical properties [42].

As can be seen, there are three different results of the tensile tests. For the UHMW PE, the decrease in tensile strength is about 13%. Since the wetting behavior is substantially increased, it should be considered to reduce the treatment intensity to minimize the impact on tensile strength. For PPS and PPTA, the decrease is significantly lower than 5%, so the use of (oxy-)fluorination as is done in these investigations is possible. For the CF, even an increase in tensile strength is observed, so CF is also feasible for (oxy-)fluorination.

## 3. Materials and Methods

### 3.1. Investigated High-Performance Fibers

Different high-performance fibers were used as substrates. The ultra-high-molecular-weight polyethylene yarn Dyneema^®^ SK65, referred to as PE, with a fineness of 21 tex and single filaments with a diameter of 18 μm, was purchased from DSM (Heerlen, The Netherlands). The used PPS fibers, Type 170 (Performance Fibers GmbH, Bad Hersfeld, Germany), have a single filament diameter of 24 μm and a fineness of 108 tex. The PPTA fibers Twaron^®^ T1000 were from Teijin Aramid (Arnhem, The Netherlands) with a single filament diameter of 12 μm and 330 tex. The carbon fiber Toho Tenax^®^ HTS 45 E23 was purchased from Toho Tenax Europe GmbH (Wuppertal, Germany). It possesses epoxy sizing, a fineness of 800 tex and a single filament diameter of 7 μm. All fibers excluding the carbon fibers were desized by washing with acetone prior to (oxy-)fluorination. The carbon fibers were thermally desized in a ceramic oven at 500 ${}^{\circ }C$ for 5 min.

### 3.2. (Oxy-)Fluorination

**Figure 6:**
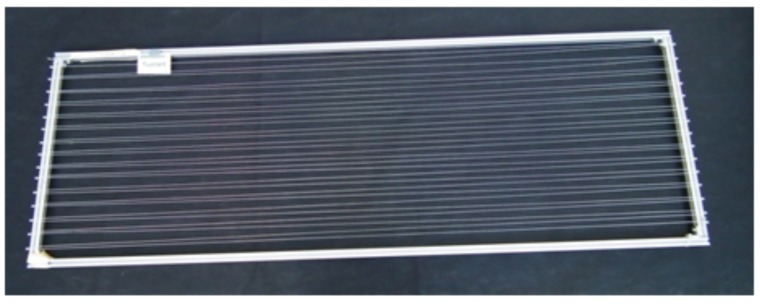
Yarn preparation as used for the (oxy-)fluorination treatment.

**Figure 7:**
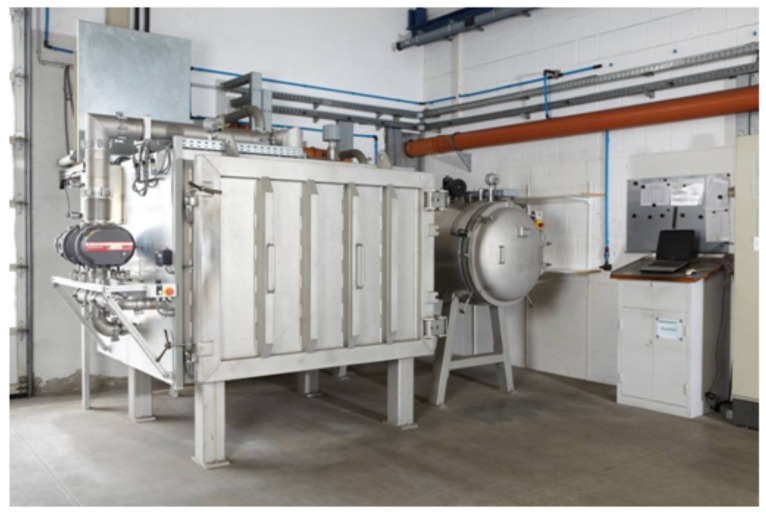
(Oxy-)fluorination chamber used for the treatment of the technical fibers, with permission from Fluor Technik System GmbH.

**Figure 8:**
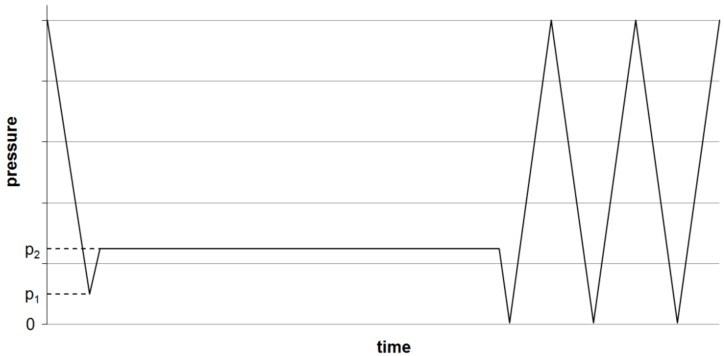
Scheme of the (oxy-)fluorination procedure. Evacuation of the reaction chamber to a pressure p1, inlet of F${}_{2}$/N${}_{2}$-mixture (10:90 vol %) till p2, maintaining pressure over the reaction time, then multiple evacuations and purging with air till all remaining fluorine is removed.

For (oxy-)fluorination, the yarns were fixed loosely to a metal frame (Figure 6) and treated in the reaction chamber (Figure 7) at room temperature. The frame has dimensions of 1.60 m × 0.55 m and allows the fluorination of about 50 m of yarn during a batch process. A typical treatment procedure is shown in Figure 8. Adjustable parameters are the fluorine concentration and the duration of the reaction. Winding sections of the yarns were cut out before subsequent characterization to eliminate unwanted influences caused by mechanical damage and inhomogeneous treatment that might occur due to hindered gas diffusion.

(Oxy-)fluorination of the polymeric fiber (UHMW PE, PPTA and PPS) was done with 2% fluorine concentration for a reaction time of 120 s (Recipe 2). Due to significantly lower impact on the carbon fiber in previous experiments, treatment conditions were intensified by increasing $F_{2}$ concentration to 5% and a longer process duration of 5 min (Recipe 6). The reacting oxygen originated from the remaining air at pressure p1 (Figure 8). After the treatment, the reaction chamber was flushed with air three times to remove left over reagents, as shown in Figure 8.

### 3.3. Contact Angle Measurements and Surface Energy Calculation

Contact angles Θ as an indicator for the wetting behavior were measured by means of single fiber tensiometry with the help of a K100SF tensiometer (Krüss GmbH, Hamburg, Germany) [46]. Here, single fibers were taken out from the yarns and immersed into distilled water (72.8 mN/m) and diiodomethane (>99%, Sigma-Aldrich Chemie GmbH, 50.8 mN/m). At least six individual fibers were measured for each sample and liquid.

Calculation of the total surface energy and the polar and disperse parts was then done according to the method of Owens, Wendt, Rabel and Kaelble [47,48,49]:

(1) $$\frac{\left(1+\cos\Theta \right)\cdot \sigma_{l}}{2\cdot \sqrt{\sigma_{l}^{D}}}=\sqrt{\sigma_{s}^{P}}\cdot \sqrt{\frac{\sigma_{l}^{P}}{\sigma_{l}^{D}}}$$

where superscripts *P* and *D* represent the polar and disperse part of the surface energies *σ*, respectively, while *l* and *s* represent the liquid and solid phases, i.e., testing liquid and fiber surface.

### 3.4. Scanning Electron Microscopy

Surface topography of treated and untreated fibers was investigated by means of a high-resolution low-voltage SEM. Therefore, samples were fixed onto an aluminum specimen holder with a conducting carbon pad. UHMW PE was sputter-deposited with Au/Pd, while the carbon fiber was coated with graphite for necessary conductivity. The micrographs were taken with a Gemini DSM 289 (Carl Zeiss AG, Jena/Oberkochen, Germany). The PPTA and the PPS samples were sputter-deposited with Pt, and these micrographs were taken using a Zeiss Ultra Plus (Carl Zeiss AG, Germany).

### 3.5. Transmission Electron Microscopy, Electron Energy-Loss Spectroscopy

The sample preparation took place at the FIB/SEM-device (NVision 40 Zeiss with focussed ionic beam(FIB)) by the use of the in situ lift-out method. The electron energy-loss near edge structure (ELNES) at the C-edge and in low-loss-region studies was performed by a Zeiss scanning transmission electron microscope (Libra 200 HR MC Cs STEM) with an in-column energy filter. The operation acceleration voltage was 200 kV. Dark field mode was used [50].

### 3.6. X-Ray Photoelectron Spectroscopy

XPS is a conventional method to estimate the surface functional groups and the surface composition. A Physical Electronics PHI 5700 ESCA System was utilized for the investigation of the carbon fibers and UHMW PE fibers to reveal the elemental composition of the surface. The instrument contains an Al K*α* anode and allows a sample depth of 9 nm with an X-ray irradiation under 250 W within a vacuum of 5 × 10^−10^ torr. In the high-resolution spectra, the single peaks for carbon (C1s) were disassembled at 281 eV to 293 eV, whereas the quantitative analyses were done with the standard PHI software. PPTA and PPS samples were analyzed using an AXIS ULTRA (Kratos Analytical Ltd., Manchester, England) under the same conditions with an Al K*α* X-ray source at 300 W.

### 3.7. Tensile Strength Tests

Textile-mechanical characterization was conducted by measuring the stress-strain behavior. The testing was carried out in accordance with ISO 3341, where polymeric yarn samples were wound around rope grips and fixed to metal grips of a Z100 tensile testing machine (Zwick GmbH, Ulm, Germany). Due to the brittleness of the carbon fibers, these were fixed using rubber grips. The measuring length was 500 mm, and the testing speed was set to 250 mm/min for all samples. Because of the relatively low breaking force for the PPS yarn, a tensile testing machine Z2.5 was used. Here, the measuring length was 250 mm because of the high maximum elongation of this yarn type. The elongation was observed by optical sensors. At least ten individual measurements were done for each sample.

## 4. Conclusions

Different, typical high-performance fibers utilized for fiber-reinforced composites were oxy-fluorinated to enhance their abilities for surface interactions. The investigation on the wetting behavior showed impressive polar surface energies for treated UHMW PE, which should result in significantly better adhesion in the composite. Using (oxy-)fluorination in combination with poly(*p*-phenylene terephthalamide) fibers, the polar part of the surface energy could not be increased. Therefore, the treatment as done in this investigation seems not to be effective for PPTA. (Oxy-)fluorination of PPS fibers leads to a significant increase in the polar part of the surface energy. Since tensile tests show that the mechanical properties are nearly the same after the fluorination, it can be concluded that this method is useful for the surface activation of PPS fibers. Compared to this, the (oxy-)fluorination of CF leads to a doubling of the polar surface energy besides an enormous increase of the measured tensile strength; however, more drastic conditions than for the organic fibers are required. The promising findings lead to further investigation into the mechanism of the (oxy-)fluorination onto the chemistry of treated carbon fibers. The treatment of organic high-performance fibers is recommended as shown for UHMW PE, but has to be examined in detail for polymers based on aromatic systems.

## Figures and Tables

**Table 1:** Contact angles (CA) and polar and disperse parts of surface energies (*σ*) before and after (oxy-)fluorination of the investigated high-performance fibers (standard deviation in parenthesis). All high-performance fibers were prepared with Recipe 2, except CF (Recipe 6).

	UHMW PE		PPS		PPTA		CF	
	Reference	Sample	Reference	Sample	Reference	Sample	Reference	Sample
CA $H_{2}O$ $\left({}^{\circ }\right)$	84.9 (1.6)	58.7 (2.8)	87.2 (1.5)	71.8 (4.9)	50.4 (3.1)	91.7 (5.3)	*68.9 (5.8)*	50.2 (5.1)
CA $CH_{2}I_{2}$ $\left({}^{\circ }\right)$	58.2 (2.7)	49.7 (2.9)	24.7 (3.6)	67.6 (2.7)	24.8 (5.8)	40.4 (5.5)	36.0 (7.1)	37.8 (6.1)
$\sigma_{pol}$ (mN/m)	4.0 (0.3)	15.2 (0.8)	0.8 (0.1)	12.0 (1.4)	15.2 (0.7)	0.7 (0.3)	7.4 (1.0)	17.5 (1.3)
$\sigma_{disp}$ (mN/m)	29.6 (0.9)	34.4 (1.2)	46.2 (1.1)	24.2 (2.4)	46.2 (1.4)	39.3 (2.4)	41.3 (2.7)	40.5 (2.3)
$\sigma_{total}$ (mN/m)	33.6 (1.2)	49.7 (1.9)	47.0 (1.3)	36.2 (3.8)	61.4 (2.2)	40.0 (2.7)	48.7 (3.7)	58.0 (3.6)

**Table 2:** Changes of chemical structure after (oxy-)fluorination shown by XPS measurements. All high-performance fibers were prepared with Recipe 2, except CF (Recipe 6).

Sample	C:O	C:N	C:F	C:S
PE reference	0.027	0.000	0.000	0.000
PE treated	0.176	0.000	0.157	0.000
PPS reference	0.115	0.000	0.000	0.040
PPS treated	0.215	0.010	0.198	0.033
PPTA reference	0.199	0.098	0.000	0.000
PPTA treated	0.251	0.042	0.241	0.000
CF reference	0.166	0.037	0.000	0.000
CF treated	0.175	0.027	0.050	0.000

**Table 3:** Stress-strain behavior of untreated and oxy-fluorinated high-performance fibers (standard deviation in parenthesis). All high-performance fibers were prepared with Recipe 2, except CF (Recipe 6).

Sample	E Modulus (GPa)	Breaking Force (N)	Tensile Strength (MPa)	Elongation at Break (%)
PE reference	82.4 (1.6)	504 (13)	2328 (61)	3.19 (0.09)
PE treated	80.0 (5.0)	440 (33)	2032 (151)	2.59 (0.13)
PPS reference	6.4 (0.1)	39.8 (1.1)	491 (16)	23.73 (0.80)
PPS treated	6.3 (0.1)	38.9 (1.5)	479 (15)	23.71 (0.76)
PPTA reference	66.4 (2.7)	601 (31)	2545 (131)	3.33 (0.15)
PPTA treated	64.7 (3.1)	600 (27)	2541 (116)	3.34 (0.12)
CF reference	136 (15.6)	592 (13)	1295 (28)	1.19 (0.16)
CF treated	124 (12.7)	683 (26)	1494 (57)	1.34 (0.07)

## Abbreviations

The following abbreviations are used in this manuscript:

CF: carbon fiber
EELS: electron energy-loss spectroscopy
ELNES: electron energy-loss near edge structure
FIB: focussed ionic beam
PEEK: polyether ether ketone
PPS: poly(*p*-phenylene sulfide)
PPTA: poly(*p*-phenylene terephthalamide)
SEM: scanning electron microscopy
(S)TEM): scanning electron microscopy
UHMW PE: ultra-high-molecular-weight polyethylene
XPS: X-ray photoelectron spectroscopy
